# Association between home and community-based services and depressive symptoms in Chinese older adults: a multilevel analysis

**DOI:** 10.1186/s12889-023-16008-9

**Published:** 2023-07-21

**Authors:** Tingke Xu, Zishuo Huang, Yucheng Huang, Shanshan Wang, Xinxin Zhang, Yaqi Hu, Yue Zhu, Dayi Cheng, Yating Fu, Xiangyang Zhang, Chun Chen

**Affiliations:** 1 School of Public Health and Management, Wenzhou Medical University, Wenzhou, Zhejiang 325000 China; 2 School of Innovation and Entrepreneurship, Wenzhou Medical University, Wenzhou, Zhejiang 325000 China; 3 First Affiliated Hospital of Wenzhou Medical University, Wenzhou, Zhejiang 325000 China; 4 Institute for County Chronic Disease Health Management Research, Wenzhou Medical University, Wenzhou Medical University, Wenzhou, Zhejiang 325000 China

**Keywords:** Depressive symptoms, Home and community-based services, Generalized hierarchical linear models, Older adults

## Abstract

**Background:**

As the phenomenon of ageing continues to intensify, home and community-based services (HCBSs) have been increasingly important in China. However, the association between HCBSs utilization and depressive symptoms in older adults in China is unclear. Consequently, this study aimed to examine the association between HCBSs utilization and depressive symptoms in Chinese older adults.

**Methods:**

This study included 7,787 older adults (≥ 60 years old) who were recruited within the framework of the 2018 China Health and Retirement Longitudinal Study (CHARLS). Depressive symptoms were assessed using the 10-item Center for Epidemiological Studies Depression Scale (CES-D-10). HCBSs utilization was assessed via the question, “What kind of HCBSs were being utilized in their community?”. Data were analyzed using binary logistic regression models and generalized hierarchical linear models (GHLM).

**Results:**

Of the 7,787 participants, 20.0% (n = 1,556) reported that they utilized HCBSs, and 36.7% (n = 2,859) were evaluated that they had depressive symptoms. After adjusting for individual- and province-level covariates, the HCBSs utilization was found to be associated with depressive symptoms (OR = 1.180, 95% CI: 1.035–1.346, *p* < 0.05). Additionally, the depressive symptoms were significantly associated with gender, residence, educational level, marital status, number of chronic diseases, self-rated health (SRH), smoking, and provincial Gross Domestic Product (GDP) per capita.

**Conclusions:**

This study found HCBSs utilization might be a protective factor against depressive symptoms in Chinese older adults. It is of utmost significance for the government to provide targeted HCBSs at the community level to address the unmet care needs of older adults, which can reduce the occurrence of negative emotions, consequently contributing to less severe depressive symptoms.

## Introduction

By the end of 2020, the number of people aged 60 years and over had surged to 264.02 million in China, which accounted for 18.70% of the overall population [1]. As the phenomenon of aging continues to intensify, the mental health of older adults, especially depression, requires more attention. Depression, one of the leading causes of the global disease burden, is a common mental health problem worldwide [2]. The World Health Organization (WHO) suggested that there is an increased incidence of depression in older adults [3], with over 10% of global older adults having experienced depressive symptoms [4]. Similarly, the prevalence rate of depressive symptoms in older adults is the highest among the general population in China [5].

In tandem with the aging population, China is also undergoing a massive social transition and changes in family structure [6, 7]. These shifts challenge the conventional family-based care arrangement for older adults, resulting in the decline of older adults’ mental health due to childlessness and an “empty nest” [8]. In addition, owning to financial burdens and dwelling preference, the institutional endowment resources cannot meet the elderly care needs of most older adults in China anymore [9]. Hence, the huge demand for home and community-based services (HCBSs) among older adults in China is evident and urgent.

HCBSs originated in Western countries and have reached a relatively high level of maturity [10, 11]. In contrast, HCBSs in China are still evolving, encompassing a variety of non-institutional services for older adults living in the community, which is provided by the government, community, and social organizations [9, 12]. HCBSs in China are primarily in line with some Western countries, except for the absence of church services and religious services, which include mainly health care services (e.g., in-home medical care services, therapy care services), social support services (e.g., entertainment, emergency call services), daily care services (e.g., meal delivery, housekeeping) [13, 14].

The positive effects of HCBSs on health status in older adults have been acknowledged, such as enhancing life satisfaction [15], promoting cognitive function [16], and reducing the risk of depressive symptoms [17]. Depressive symptoms are a prevalent and serious mental health issue that deserve attention [2]. However, as mentioned in the previous study’s limitations [17], only the relationship between depressive symptoms and HCBSs provision based on the Chinese Longitudinal Healthy Longevity Survey (CLHLS), rather than the utilization of HCBSs, was examined, which may have led to a misjudgment of the role of HCBSs on older adults’ depressive symptoms. Moreover, the health status of older adults was confirmed to be a hierarchical structure, meaning that individuals were nested within specific provinces [18]. Thus, individual- and province-level factors would affect the health status. However, the previous studies conducted a regression analysis to examine the effect of HCBSs on older adults’ health only adjusting individual-level covariates [15–17]. In contrast, the province-level covariates might bias these estimations.

Therefore, to fill these research gaps, the current study assessed the HCBSs utilization and depressive symptoms in Chinese older adults, and examined their relationship with generalized hierarchical linear models (GHLM) based on the 2018 China Health and Retirement Longitudinal Study (CHARLS). The findings of this study could shed light on the future policy implementation on the development of HCBSs in China.

## Methods

### Study participants

Data were obtained from the fourth wave of survey data from the CHARLS, which is a survey of middle-aged and older adults in China based on a sample of households with members aged 45 years or above. With the purpose of establishing a high-quality public database, it contained a wide range of information, ranging from socioeconomic status to health conditions, needed for scientific research on middle-aged and older adults [19, 20]. To ensure sample representative, the survey followed strict randomization procedures and used a multi-stage sampling method. When sampling county and rural administrative units, a Probability-Proportional-to-Size (PPS) sampling method was adopted [21].

In this study, participants were excluded if they (1) were younger than 60 years old, or lacked data regarding (2) utilization of HCBSs, (3) 10-item Center for Epidemiological Studies Depression (CES-D-10), or (4) values in the main variables. A total of 19,816 participants completed the CHARLS in 2018. Crucially, 8,762 participants aged lower than 60 years, 35 participants lacking utilization of HCBSs data, 2,668 participants lacking CES-D-10 data, 530 participants with Memory-Related Disease or Emotional Problems, and 34 participants lacking values in main variables were excluded. Accordingly, the final study sample consisted of 7,787 participants.

### Measurement

#### Dependent variable

Depressive symptoms were assessed using the CES-D-10 [22]. Ten of 20 items from the original version of the scale were grouped to the shortened CES-D-10 scale by its internal correlations [23]. This scale was widely used in older adults and showed high reliability and validity among Chinese older adults [24]. The CES-D-10 was comprised of ten questions about depression, and the answers included four options: (1) rarely, (2) some days (1–2 days per week), (3) occasionally (3–4 days per week), and (4) most of the time (5–7 days per week). Among the ten questions, eight questions were negative statements, and two questions were positive statements. The answers were recorded as 0 (rarely) to 3 (most of the time) for the negative questions and were recorded as 3 (rarely) to 0 (most of the time) for the positive questions. The total score ranged from 0 to 30 points. A score ≥ 10 in the CES-D-10 was considered to indicate depressive symptoms, as validated by previous studies [23].

#### Independent variable

HCBSs utilization was measured by asking participants, “Have you ever received the following home and community care services?” The response options included: (a) day care centers, nursing homes, senior dining tables, etc.; (b) regular physical examinations; (c) on-site visits; (d) family beds; (e) community nursing; (f) health management; (g) entertainment. If participants used one or more HCBS, they were classified as HCBSs utilization group, otherwise they were classified as HCBSs non-utilization group.

#### Covariates

Covariates were considered at individual- and province-levels [25–28]. Individual-level covariates included sociodemographic characteristics, health status, and lifestyle. Sociodemographic characteristics included age, gender, marital status, educational level, residence, income, and health insurance; health status included the number of chronic diseases and self-rated health (SRH); lifestyle factors included smoking, drinking, and exercising. Further, the province-level covariates included 2018 provincial Gross Domestic Product (GDP) per capita and Number of beds in medical institutions per 10,000 persons, reflecting economic and medical resources respectively.

### Statistical analysis

Descriptive statistics were used to analyze characteristics of the study participants. Binary logistic regression was used to compare depressive symptoms risk across groups based on the categorical variables. GHLM were constructed using mixed model procedures, given that the data had a hierarchical structure composed of individuals (Level 1) nested within provinces (Level 2). These models not only estimated fixed effects of individual- and province-level factors on depressive symptoms, but also tested for the variance components for the random intercepts, representing the differences in older adults’ depressive symptoms at the province-level. Data were analyzed using SPSS version 26.0. The level of significance was set at *p* < 0.05.

## Results

Table 1 presents the basic characteristics of the total sample included in this study. The final sample included 7,787 participants from 28 provinces, representing approximately 264 million Chinese residents aged ≥ 60 years. Of the 7,787 participants, 20.0% (n = 1,556) utilized HCBSs, and 36.7% (n = 2,859) with depressive symptoms. A majority of the respondents were male (n = 4,034, 51.8%), aged 60–69 (n = 5,074, 65.2%), partnered (n = 6,378, 81.9%), with an educational level of elementary school (n = 3,580, 46.0%), living in rural areas (n = 5,627, 72.3%), without income (n = 6,651, 85.4%), and without health insurance (n = 7,584, 97.4%). Additionally, a significant number of the respondents did not drink (n = 5,127, 65.8%) and smoke (n = 5,582, 71.7%), exercised (n = 7,041, 90.4%), had fair SRH (n = 3,897, 50.1%), and had three or more chronic diseases (n = 3,106, 39.9%). Most of the participants lived in the provinces with the third quartile of GDP per capita (n = 2,396, 30.8%) and provinces with the second quartile of Number of beds in medical institutions per 10,000 persons (n = 2,221, 28.5%).

**Table 1:** Depressive symptoms by individual- and province-level variables in Chinese older adults in 2018

Variable	N (%)	Depressive Symptoms		OR (95% CI)	*p-*value
		No	Yes		
**Total**	7,787 (100.0)	4,928 (63.3)	2,859 (36.7)		
**Individual level**					
Utilization of HCBSs					
Yes	1,556 (20.0)	1,028 (66.1)	528 (33.9)	Ref	
No	6,231 (80.0)	3,900 (62.6)	2,331 (37.4)	1.164 (1.035, 1.308)	**0.011**
Gender					
Male	4,034 (51.8)	2,832 (70.2)	1,202 (29.8)	Ref	
Female	3,753 (48.2)	2,096 (55.8)	1,657 (44.2)	1.863 (1.697, 2.045)	**< 0.001**
Age					
60–69	5,074 (65.2)	3,245 (64.0)	1,829 (36.0)	Ref	
70–79	2,252 (28.9)	1,392 (61.8)	860 (38.2)	1.096 (0.989, 1.214)	0.079
80–	461 (5.9)	291 (63.1)	170 (36.9)	1.036 (0.851, 1.263)	0.722
Marital status					
Partnered	6,378 (81.9)	4,158 (65.2)	2,220 (34.8)	Ref	
Single	1,409 (18.1)	770 (54.6)	639 (45.4)	1.554 (1.383, 1.747)	**< 0.001**
Educational level					
Illiterate	2,011 (25.8)	1,072 (53.3)	939 (46.7)	Ref	
Elementary school	3,580 (46.0)	2,197 (61.4)	1,383 (38.6)	0.719 (0.644, 0.803)	**< 0.001**
Middle school	1,361 (17.5)	992 (72.9)	369 (27.1)	0.425 (0.366, 0.492)	**< 0.001**
High school or higher	835 (10.7)	667 (79.9)	168 (20.1)	0.288 (0.238, 0.348)	**< 0.001**
Residence					
Urban	1,630 (20.9)	1,211 (74.3)	419 (25.7)	Ref	
Urban-rural continuum	530 (6.8)	361 (68.1)	169 (31.9)	1.353 (1.093, 1.676)	**0.006**
Rural	5,627 (72.3)	3,356 (59.6)	2,271 (40.4)	1.956 (1.729, 2.212)	**< 0.001**
Income					
Yes	1,136 (14.6)	848 (74.6)	288 (25.4)	Ref	
No	6,651 (85.4)	4,080 (61.3)	2,571 (38.7)	1.855 (1.609, 2.140)	**< 0.001**
Health insurance					
Yes	203 (2.6)	107 (52.7)	96 (47.3)	Ref	
No	7,584 (97.4)	2,763 (36.4)	4,821 (63.6)	0.639 (0.483, 0.845)	**0.002**
Number of chronic diseases					
0	1,186 (15.2)	931 (78.5)	255 (21.5)	Ref	
1	1,778 (22.8)	1,257 (70.7)	521 (29.3)	1.513 (1.274, 1.797)	**< 0.001**
2	1,717 (22.1)	1,097 (63.9)	620 (36.1)	2.063 (1.741, 2.446)	**< 0.001**
≥ 3	3,106 (39.9)	1,463 (47.1)	1,643 (52.9)	3.251 (2.783, 3.798)	**< 0.001**
SRH					
Very good	860 (11.0)	721 (83.8)	139 (16.2)	Ref	
Good	917 (11.8)	726 (79.2)	191 (20.8)	1.365 (1.072, 1.738)	**0.012**
Fair	3,897 (50.1)	2,651 (68.0)	1,246 (32.0)	2.438 (2.009, 2.959)	**< 0.001**
Bad	1,635 (21.0)	686 (42.0)	949 (58.0)	7.176 (5.837, 8.821)	**< 0.001**
Very bad	478 (6.1)	144 (30.1)	334 (69.9)	12.031 (9.214, 15.709)	**< 0.001**
Smoking					
Yes	2,205 (28.3)	1,472 (66.8)	733 (33.2)	Ref	
No	5,582 (71.7)	3,456 (61.9)	2,126 (38.1)	1.235 (1.114, 1.370)	**< 0.001**
Drinking					
Yes	2,660 (34.2)	1,868 (70.2)	792 (29.8)	Ref	
No	5,127 (65.8)	3,060 (59.7)	2,067 (40.3)	1.593 (1.441, 1.761)	**< 0.001**
Exercising					
Yes	7,041 (90.4)	4,518 (64.2)	2,523 (35.8)	Ref	
No	746 (9.6)	410 (55.0)	336 (45.0)	1.468 (1.260, 1.709)	**< 0.001**
**Province level**					
GDP per capita					
Q1	1,706 (21.9)	1,013 (59.4)	693 (40.6)	Ref	
Q2	2,296 (29.5)	1,362 (59.3)	934 (40.7)	1.002 (0.882, 1.139)	0.970
Q3	2,396 (30.8)	1,528 (63.8)	868 (36.2)	0.830 (0.731, 0.943)	**0.004**
Q4	1,389 (17.8)	1,025 (73.8)	364 (26.2)	0.519 (0.445, 0.605)	**< 0.001**
Number of beds in medical institutions per 10,000 persons					
Q1	2,062 (26.5)	1,281 (62.1)	781 (37.9)	Ref	
Q2	2,221 (28.5)	1,529 (68.8)	692 (31.2)	0.742 (0.654, 0.842)	**< 0.001**
Q3	1,701 (21.8)	1,090 (64.1)	611 (35.9)	0.919 (0.805, 1.050)	0.216
Q4	1,803 (23.2)	1,028 (57.0)	775 (43.0)	1.237 (1.087, 1.407)	**< 0.001**

*Note*: Boldface indicates statistical significance (*p* < 0.05)

Further, we analyzed the odds ratio (OR) for older adults with depressive symptoms (vs. non-depressive symptoms) for each predictor using binary logistic regression. The analysis showed that older adults who did not utilize HCBSs (OR = 1.164, 95% CI: 1.035–1.308, *p* = 0.011) had a greater risk of depressive symptoms than those who utilized HCBSs. In addition, older adults who were female, single, illiterate, living in rural areas, without income, with health insurance, with chronic diseases, with worse SRH, and who did not smoke, drink, or exercise had greater risk of depressive symptoms. For the province-level characteristics, older adults living in provinces with the fourth quartile of GDP per capita were less likely to suffer from depressive symptoms, and provinces with fourth quartile of Number of beds in medical institutions per 10,000 persons were more likely to suffer from depressive symptoms.

Table 2 summarizes the relationship between HCBSs utilization and depressive symptoms. Further, Model 1 identifies significant variations between provinces in the empty model (i.e., model without explanatory covariates included). The HCBSs utilization was significantly associated with depressive symptoms (OR = 1.149, 95% CI: 1.019–1.296, *p* < 0.05) in Model 2, which included HCBSs utilization as the only predictor. In Model 3, utilization of HCBSs was significantly associated with depressive symptoms (OR = 1.186, 95% CI: 1.040–1.352, *p* < 0.05) after controlling for other individual-level covariates. In Model 4, depressive symptoms were greatly associated with HCBSs utilization (OR = 1.180, 95% CI: 1.035–1.346, *p* < 0.05) after adjusting for individual- and province-level variables. Older adults utilizing HCBSs were more likely to have a lower risk of depressive symptoms. Several covariates (i.e., gender, marital status, educational level, residence, number of chronic diseases, SRH, and smoking) were associated with depressive symptoms. In addition, provincial GDP per capita emerged as significant predictors of depressive symptoms.

**Table 2:** HCBSs utilization associated with depressive symptoms in Chinese older adults in 2018

Fixed Effects	Model 1 OR (95% CI)	Model 2 OR (95% CI)	Model 3 OR (95% CI)	Model 4 OR (95% CI)
Individual level				
HCBSs (ref: yes)		**1.149** ^*^ **(1.019, 1.296)**	**1.186** ^*^ **(1.040, 1.352)**	**1.180** ^*^ **(1.035, 1.346)**
Gender (ref: male)			**1.700** ^***^ **(1.489, 1.941)**	**1.702** ^***^ **(1.490, 1.943)**
Age (ref: 60–)				
70–			0.916 (0.814, 1.030)	0.917 (0.815, 1.031)
80–			0.891 (0.710, 1.118)	0.896 (0.713, 1.125)
Marital status (ref: partnered)			**1.301** ^***^ **(1.137, 1.488)**	**1.297** ^***^ **(1.133, 1.484)**
Educational level (ref: illiterate)				
Elementary school			0.896 (0.787, 1.021)	0.893 (0.784, 1.017)
Middle school			**0.668** ^***^ **(0.560, 0.797)**	**0.664** ^***^ **(0.557, 0.792)**
High school or higher			**0.499** ^***^ **(0.400, 0.624)**	**0.500** ^***^ **(0.400, 0.625)**
Residence (ref: urban)				
Urban-Rural continuum			1.188 (0.942, 1.497)	1.184 (0.939, 1.493)
Rural			**1.593** ^***^ **(1.379, 1.840)**	**1.593** ^***^ **(1.379, 1.841)**
Income (ref: yes)			**1.180** ^**^ **(1.006, 1.385)**	1.166 (0.993, 1.369)
Health insurance (ref: yes)			0.809 (0.594, 1.101)	0.809 (0.594, 1.103)
Number of chronic diseases (ref: 0)				
1			**1.214** ^*^ **(1.011, 1.458)**	**1.215** ^*^ **(1.012, 1.459)**
2			**1.517** ^***^ **(1.263, 1.822)**	**1.514** ^***^ **(1.260, 1.818)**
≥ 3			**1.895** ^***^ **(1.591, 2.258)**	**1.887** ^***^ **(1.584, 2.248)**
SRH (ref: very good)				
Good			1.253 (0.975, 1.610)	1.249 (0.972, 1.606)
Fair			**1.980** ^***^ **(1.615, 2.427)**	**1.973** ^***^ **(1.609, 2.419)**
Bad			**4.660** ^***^ **(3.732, 5.819)**	**4.637** ^***^ **(3.713, 5.791)**
Very bad			**7.664** ^***^ **(5.762, 10.194)**	**7.628** ^***^ **(5.734, 10.148)**
Smoking (ref: yes)			**0.826** ^**^ **(0.723, 0.944)**	**0.826** ^**^ **(0.723, 0.944)**
Drinking (ref: yes)			1.024 (0.907, 1.156)	1.026 (0.908, 1.159)
Exercising (ref: yes)			1.179 (0.993, 1.399)	1.186 (0.999, 1.408)
GDP per capita (ref: Q1)				
Q2				1.076 (0.786, 1.474)
Q3				0.899 (0.662, 1.222)
Q4				**0.573** ^**^ **(0.402, 0.818)**
Number of beds in medical institutions per 10,000 persons (ref: Q1)				
Q2				0.856 (0.627, 1.170)
Q3				0.930 (0.676, 1.286)
Q4				1.067 (0.762, 1.495)
**Random Effects** (intercept only)				
Variance Component	**0.183** ^**^	**0.184** ^**^	**0.093** ^**^	**0.058** ^*^

*Note*: Boldface indicates statistical significance (^*^p < 0.05, ^**^p < 0.01, ^***^p < 0.001)

## Discussion

To the best of our knowledge, this is the first study to explore the association between HCBSs utilization and depressive symptoms in Chinese older adults using nationally representative survey data with GHLM. Moreover, this study assessed the association between individual- and provincial-level covariates and depressive symptoms in older adults. Results showed that older adults who utilized HCBSs were less likely to suffer from depressive symptoms, even after adjusting for individual- and province-level covariates.

In the early 1980s, HCBSs provision systems were first established in the United States and Denmark to match the demands of older adults [10, 11]. However, the implementation of HCBSs in Western countries occurred earlier than in China. In response to the aging population, China did not strongly encourage the implementation of HCBSs to protect the health of older adults until 2008. Due to the implementation and rapid promotion of national policies, from 2008 to 2018, the provision rate of HCBSs has increased dramatically from 26.1 to 62.0% in China [29]. However, the utilization rate of HCBSs was just 20.0% in 2018, which was significantly lower than the provision rate of HCBSs in China. This gap may be due to the provision of HCBSs that cannot precisely match the demand of HCBSs among Chinese older adults.

The prevalence of depressive symptoms in older adults aged 60 years and above was 36.7% in 2018, which was significantly higher than that in 2015 [25], despite the same measurement and cut-off points being used. In traditional Chinese society, positive family relations are beneficial to mental health and act as a social security system for older adults [30]. However, rapid industrialization and urbanization have shifted these traditional arrangements. Nowadays, older adults are less likely to live with younger generations and interact with them. This has direct implications on access to social care and financial security and even affects the mental health of older adults [31, 32].

Previous studies on depressive symptoms in older adults focused only on individual factors [27, 33, 34], leading to scientific bias in the research. In this study, GHLM was used to comprehensively consider the influence of individual- and province-level factors, making the results more reliable. The present study found that individual characteristics, including gender, marital status, educational level, residence, number of chronic diseases, SRH, and smoking might be associated with depressive symptoms, which is in line with previous research [35–41]. Notably, the provincial factor (GDP per capita) was also found to be an important factor being associated with depressive symptoms. Older adults living in provinces with higher economic levels were less likely to suffer from depressive symptoms. This is mainly due to good financial status, which could guarantee a certain amount of public expenditure to build a superior older adults’ care service system to reduce loneliness and depressive symptoms [42]. However, this study found no significant association between individual factors including age, income, health insurance, drinking, exercising, and provincial factor (Number of beds in medical institutions per 10,000 persons) and depressive symptoms.

Our findings supported the hypothesis that HCBSs utilization was associated with depressive symptoms in the aging population in China. Firstly, the family’s ability to provide older adults’ care has been reduced following demographic shifts and socioeconomic changes [43], and older adults may feel a sense of loneliness or disappointment if they fail to receive care from their children [44]. However, the daily care services of HCBSs, such as day care centers, nursing homes, senior dining tables, etc., could be an alternative to accompanying children, reduce feelings of loneliness and marginalization, and prevent older adults from depressive symptoms. Secondly, HCBSs included not only daily care services, but also health care services, such as regular physical examinations, onsite visits, family beds, community nursing, and health management, which are significant approaches to promote somatic symptoms and alleviate depressive symptoms in older adults to a certain extent [45]. Thirdly, some entertainment activities organized by the volunteers or self-organized by the older adults through community spaces and equipment are also an important part of HCBSs and could provide an opportunity to interact with others and reduce negative affect among older adults who live alone at home [46]. Thus, providing targeted HCBSs at the community level could meet elderly care needs and may reduce the incidence of adverse emotions and consequently lower the risk of depressive symptoms.

This study aims to enhance our understanding of the role of HCBSs in benefiting mental health. To ease the depressive symptoms in community-dwelling older adults, it is expected that more financial and human resources will be allocated to extend the coverage of HCBSs further and make them more accessible to older adults in China, especially to those who are female, single, with chronic diseases, with worse SRH, not smoking, and living in a rural and the area with the lag of economic development. What’s more, policymakers should not only strive to improve the quantity of HCBSs but also lay more emphasis on their quality. In particular, HCBSs should be provided pointedly in terms of the preferences of distinct types of older adult to match the provision and demand of HCBSs, thus truly improving the utilization of HCBSs.

## Limitation

There are also some limitations in this study. First, the data regarding HCBSs utilization was not included in the CHARLS until 2018, so we can only conduct the cross-sectional study. Hence the causal relationship between the utilization of HCBSs and depressive symptoms was not examined in this study. Prospective studies should further investigate the relationship as long as longitudinal data is available. Second, the associations between specific types of HCBSs, such as daily care, health care, and in Chinese older adults were not measured in this study. Further research is needed to examine the associations between specific types of HCBS and depressive symptoms. Third, CHARLS might exist some missing cases regarding depressive symptoms of the participants, resulting in selection bias. In the future, the surveys we conduct should take this issue fully into account.

## Conclusions

This study shows that depressive symptoms were associated with HCBSs utilization in China applying GHLM to partly address potential selection bias issues. Better depressive symptoms are more prevalent among Chinese older adults with HCBSs utilization. The findings of this study provide a better understanding about the association between HCBSs and depressive symptoms in Chinese older adults and imply that government should strive to extend HCBSs coverage and promote the quality of these services to match the demand of HCBSs, thus improving older adults’ mental health.

## Data Availability

All data used in this study were stored at http://charls.pku.edu.cn/ and available upon request.

## Abbreviations

CES-D-10: 10-item Center for Epidemiological Studies Depression Scale
CHARLS: China Health and Retirement Longitudinal Study
GDP: Gross Domestic Product
GHLM: Generalized Hierarchical Linear Models
HCBSs: Home and Community-Based Services
OR: Odds Ratio
PPS: Probability-Proportional-to-Size
SRH: Self-Rated Health
